# Electrocardiographic Predictors of High-Risk Patent Foramen Ovale Anatomy Defined by Transesophageal Echocardiography

**DOI:** 10.3390/jcm14207138

**Published:** 2025-10-10

**Authors:** Semih Kalkan, Muhammet Tekin

**Affiliations:** Department of Cardiology, Basaksehir Cam Sakura City Hospital, Istanbul 34480, Türkiye; muhteki@gmail.com

**Keywords:** cryptogenic stroke, patent foramen ovale, electrocardiography, transesophageal echocardiography, high-risk anatomy, right-to-left shunt, crochetage R wave

## Abstract

**Background**: Patent foramen ovale (PFO) is a common finding linked to cryptogenic stroke. Transesophageal echocardiography identifies high-risk anatomical features, but it remains unknown whether electrocardiography (ECG) may distinguish between high-risk and low-risk PFO anatomies. **Methods**: This retrospective single-center study included 207 consecutive patients (median age 45 years; 46.9% male) who underwent percutaneous PFO closure between January 2021 and June 2025. Patients were stratified into low-risk (score 0–1, *n* = 46), and high-risk (score 2–5, *n* = 161) groups using the Nakayama risk score. Baseline 12-lead ECGs were analyzed for crochetage R wave, right bundle branch block, RSR’ pattern, and T-wave abnormalities. Clinical, laboratory, and echocardiographic data were systematically evaluated. **Results**: High-risk patients more frequently exhibited crochetage R waves (40.4% vs. 17.4%, *p* = 0.004) and spontaneous Doppler shunting (53.3% vs. 31.0%, *p* = 0.010). Crochetage R wave strongly correlated with the presence of a large right-to-left shunt (≥20 bubbles: 97.2% vs. 82.0%, *p* = 0.002), reinforcing its pathophysiological significance. The presence of a crochetage R wave was independently associated with high-risk PFO anatomy (OR: 32.4; 95% CI: 2.64–397.7; *p* = 0.007). In addition, spontaneous Doppler shunting (OR: 5.4; 95% CI: 1.1–26.4; *p* = 0.039) and absence of lipomatous hypertrophy (OR: 0.10; 95% CI: 0.01–0.71; *p* = 0.022) were independent predictors of high-risk PFO anatomy. **Conclusions**: In patients with PFO, ECG changes such as the crochetage R wave are driven by anatomical risk features and shunt magnitude and may aid noninvasive risk stratification in cryptogenic stroke.

## 1. Introduction

Patent foramen ovale (PFO) is a frequent congenital interatrial communication, present in approximately 20–25% of the general population. While often incidental, PFO has been strongly linked to cryptogenic stroke through paradoxical embolism [1]. Importantly, not all PFOs carry the same clinical significance; only those with specific high-risk anatomical features are considered pathogenic and more likely to cause ischemic events [2].

Transesophageal echocardiography (TEE) with contrast is regarded as the gold standard for diagnosing PFO and identifying high-risk anatomies [3]. These include long-tunnel PFOs, large septal separation, atrial septal aneurysm, hypermobile septum, prominent Eustachian valve or Chiari network, and large right-to-left shunt during Valsalva maneuver [2]. Accurate recognition of such features is essential, as patients with high-risk PFOs are most likely to benefit from percutaneous closure [4].

Electrocardiography (ECG), as a widely accessible and noninvasive modality, has been investigated as a potential adjunctive tool for identifying PFO-related substrates. A characteristic “crochetage” pattern—an M-shaped notch on the ascending limb or zenith of the R wave in inferior leads—was first described in patients with atrial septal defects and later observed in cryptogenic stroke patients with PFO [5,6]. Several studies have confirmed an association between crochetage R waves and PFO, with improved predictive accuracy when combined with right bundle branch block (RBBB) [7]. However, findings have not been entirely consistent. For example, Belvís et al. reported that crochetage and RBBB were more frequent in patients with PFO-related stroke, but the differences did not reach statistical significance, suggesting that no single ECG marker reliably identifies PFO in this setting [8]. Taken together, the available evidence points toward a potential link between surface ECG features and PFO anatomy, but with conflicting results across cohorts. Moreover, most prior studies compared PFO-positive versus PFO-negative stroke patients, without stratifying according to anatomical risk. Whether specific ECG signatures correspond to TEE-defined high-risk versus low-risk PFO anatomy in patients undergoing percutaneous closure has not been systematically explored.

The present study aims to investigate ECG findings in patients with cryptogenic stroke and PFO who subsequently underwent transcatheter closure, stratified by TEE-defined anatomical risk. By exploring the relationship between risk anatomy and surface ECG features, this study seeks to refine noninvasive risk stratification and provide new insights into the pathophysiological mechanisms linking PFO to ischemic stroke.

## 2. Methodology

### 2.1. Study Design and Population

This retrospective single-center study included 207 consecutive patients who underwent percutaneous closure of a PFO between January 2021 and June 2025.

Inclusion criteria were as follows:

1. Patients who underwent percutaneous PFO closure;
2. Age ≥ 18 years;
3. Availability of complete medical, clinical, and laboratory records;
4. TEE performed as part of the diagnostic evaluation.

Exclusion criteria included the following:

1. Inadequate echocardiographic or ECG image quality;
2. Known pulmonary hypertension;
3. Heart failure with either reduced or preserved ejection fraction;
4. Severe organic or secondary valvular disease;
5. Pre-excitation syndromes (e.g., Wolff–Parkinson–White);
6. Systemic illnesses likely to confound outcomes (e.g., advanced hepatic dysfunction, active infection, or malignancy).

A total of 234 patients were screened for eligibility. Of these, 27 were excluded: 4 due to incomplete medical records or missing laboratory/clinical data, 5 because of inadequate echocardiographic or ECG image quality, 3 with pulmonary hypertension, 5 with heart failure (both systolic and diastolic), 4 with severe organic or secondary valvular disease, 2 with pre-excitation syndromes, and 4 with systemic illnesses that could confound study outcomes. The final study population consisted of 207 patients, stratified according to the PFO risk score into low-risk (score 0–1, *n* = 46) and high-risk (score 2–5, *n* = 161) groups. A detailed flowchart of patient selection is presented in Supplemental Figure S1.

The study protocol was approved by the local institutional ethics committee, and all procedures adhered to the principles outlined in the Declaration of Helsinki. All identifiable patient information was anonymized to ensure privacy and compliance with ethical and legal regulations.

### 2.2. Electrocardiographic Evaluation

**Figure 1:**
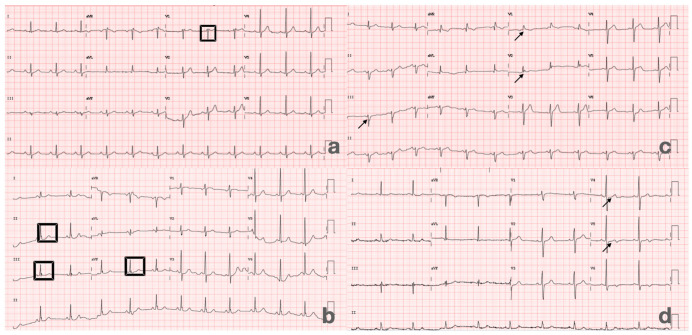
Representative ECG patterns observed in patients with PFO. (**a**) RSR’ pattern in lead V1 (in square), characterized by terminal R’ deflection suggestive of right ventricular conduction delay. (**b**) Incomplete RBBB with QRS duration < 120 ms, showing rSR’ morphology (arrow) in right precordial leads. (**c**) Crochetage R wave, defined by a notched or “saw-tooth” appearance of the R wave in inferior leads (highlighted in squares). (**d**) Defective T wave morphology, including abnormal inversion or flattening, consistent with repolarization disturbance (arrows).

Standard 12-lead ECGs were obtained at baseline for all patients. Tracings were independently reviewed by two experienced cardiologists who were blinded to clinical and echocardiographic data. Specific ECG parameters of interest included the presence of crochetage R wave in inferior leads (II, III, aVF), incomplete or complete RBBB, RSR’ pattern, and T-wave abnormalities (Figure 1a–d).

Patients with pre-excitation syndromes (e.g., Wolff–Parkinson–White) were excluded to avoid confounding of ECG interpretation. Interobserver disagreements were resolved by consensus, and parasitic waveforms or artifacts were carefully distinguished from true pathological findings to ensure accuracy of interpretation.

### 2.3. Echocardiographic Assessment

**Figure 2:**
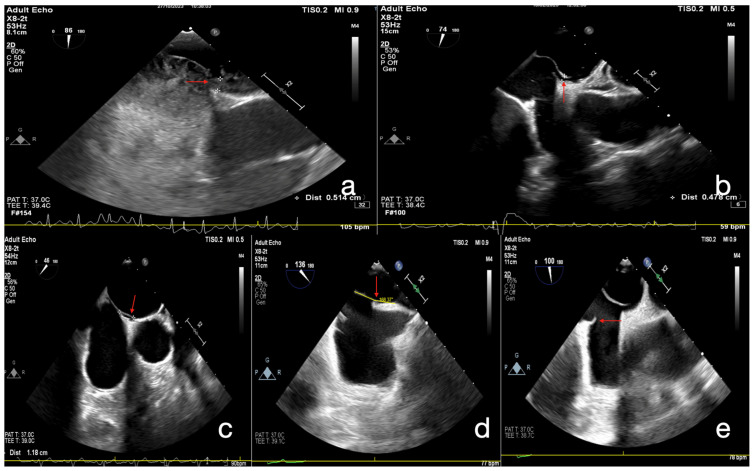
Representative echocardiographic features of high-risk PFO anatomy. (**a**) Contrast echocardiography demonstrating a large (>20) right-to-left bubble shunt during Valsalva maneuver. (direction indicated by an arrow). (**b**) Atrial septal aneurysm with marked septal excursion > 10–15 mm into either atrium (arrow). (**c**) PFO tunnel length, visualized as an extended overlap between septum primum and septum secundum (arrow). (**d**) Inferior vena cava (IVC) angle measuring 12°, with flow directed toward the interatrial septum (arrow). (**e**) Prominent Eustachian valve (arrow) at the junction of the IVC and right atrium, channeling blood toward the PFO.

All patients underwent transthoracic echocardiography (TTE) as the initial imaging modality. This was followed by contrast transthoracic echocardiography (cTTE) with agitated saline injection, performed both at rest and during the Valsalva maneuver to assess right-to-left shunting. A positive bubble study was documented in all patients prior to TEE. In cases with suspected PFO, TEE was performed, including real-time three-dimensional (RT-3D) imaging when indicated [9]. Morphological and functional parameters of the interatrial septum and PFO were systematically evaluated, including atrial septal aneurysm (ASA), Chiari network/Eustachian valve, tunnel length, tunnel width, and the IVC–PFO tunnel angle. Right-to-left shunting was assessed using agitated saline contrast both at rest and during Valsalva maneuver, and categorized according to bubble count. The presence of spontaneous right-to-left shunting on color Doppler was also documented. All examinations were performed in accordance with current echocardiographic guidelines. To stratify patients according to anatomical severity, we applied the high-risk PFO scoring system proposed by Nakayama et al. [2]. This model assigns one point for each of the following echocardiographic features (Figure 2a–e): long-tunnel PFO (≥10 mm), hypermobile interatrial septum, presence of a prominent Eustachian valve or Chiari’s network, large right-to-left shunt during Valsalva maneuver, and low-angle PFO (≤10° from the inferior vena cava). A total score ≥ 2 points was defined as high-risk PFO, whereas scores of 0–1 were classified as low-risk.

### 2.4. Laboratory Analysis

Venous blood samples were collected at baseline and analyzed in the institutional central laboratory. Hematological parameters included white blood cell count, hemoglobin, and platelet count. Biochemical indices included fasting glucose, blood urea nitrogen (BUN), creatinine, estimated, lipid profile (total cholesterol, LDL, HDL, triglycerides), HbA1c, and homocysteine. All assays were performed using standardized automated methods with internal quality control.

### 2.5. Statistical Analysis

All statistical analyses were performed using SPSS software version 29.0 (IBM Corp., Armonk, NY, USA). Categorical variables were summarized as frequencies and percentages, while continuous variables were expressed as mean ± standard deviation (SD) or as median with minimum and maximum values, according to distribution. The normality of continuous data was assessed with the Kolmogorov–Smirnov test. Group comparisons for categorical variables were conducted using the chi-square test. For continuous variables, the independent samples *t*-test was applied when normal distribution assumptions were satisfied; otherwise, the Mann–Whitney *U* test was used. Univariate logistic regression analysis was initially performed to identify potential predictors of high-risk PFO anatomy. Variables with a *p* value < 0.10 in univariate testing were subsequently entered into a multivariate logistic regression model using a backward stepwise elimination approach. Odds ratios (ORs) with corresponding 95% confidence intervals (CIs) were reported. A two-sided *p* value < 0.05 was considered statistically significant.

### 2.6. Study Outcomes

The primary outcome of this study was the identification of ECG markers predictive of TEE-defined high-risk PFO anatomy in patients undergoing percutaneous closure. Secondary outcomes included the evaluation of clinical, laboratory, and additional ECG parameters in relation to PFO risk classification.

## 3. Results

The study included 207 patients stratified according to PFO risk score groups: low-risk (score 0–1, *n* = 46) and high-risk (score 2–5, *n* = 161). The median age of the overall cohort was 45.0 years (IQR 37.0–52.0), with patients in the low-risk group being slightly older than those in the high-risk group (46.5 [41.8–53.0] vs. 44.0 [35.5–50.0] years, *p* = 0.042). Male sex accounted for 46.9% of the population, with a similar distribution across groups (37.0% vs. 49.7%, *p* = 0.127). With respect to medical therapy at baseline, ACEi/ARB use was reported in 28.5% (34.7% vs. 26.7%, *p* = 0.183), beta-blocker use in 38.6% (32.6% vs. 40.9%, *p* = 0.392), aspirin in 20.2% (28.2% vs. 18.0%, *p* = 0.188), and statins in 14.0% (21.7% vs. 11.8%, *p* = 0.141). Stroke was the most common closure indication (95.7%), followed by TIA (4.3%), with no difference between groups (*p* = 0.419 and *p* = 0.317, respectively). Comorbidities were generally infrequent and balanced between groups: diabetes mellitus 16.5% (20.0% vs. 15.5%, *p* = 0.475), hypertension 32.0% (40.0% vs. 29.8%, *p* = 0.195), coronary artery disease 9.7% (8.9% vs. 9.9%, *p* = 1.000), prior stroke 6.3% (8.7% vs. 5.6%, *p* = 0.491), smoking 17.7% (8.9% vs. 20.3%, *p* = 0.078), hyperlipidemia 22.1% (13.3% vs. 24.5%, *p* = 0.110), migraine 3.9% (0% vs. 5.0%, *p* = 0.204), deep vein thrombosis 2.9% (0% vs. 3.7%, *p* = 0.342), and atrial fibrillation 1.9% (0% vs. 2.5%, *p* = 0.577, Table 1). Functional echocardiographic and ECG findings are summarized in Table 2. High-risk patients more frequently exhibited atrial septal aneurysm/hypermobile septum (49.4% vs. 4.4%, *p* < 0.001) and Chiari/Eustachian valve (25.0% vs. 2.2%, *p* < 0.001). A large right-to-left shunt (≥20 bubbles) was markedly more common in the high-risk group (95.0% vs. 58.7%, *p* < 0.001). Lipomatous hypertrophy was more prevalent in low-risk group (*n* = 6, 13.04% vs. *n* = 11, 6.8%, *p* = 0.023). Spontaneous color Doppler shunting was observed more often in high-risk patients (53.3% vs. 31.0%, *p* = 0.010), while shunting at rest and Valsalva were comparable between groups (*p* = 0.659 and *p* = 0.590, respectively). Regarding *ECG* findings, crochetage R wave was significantly more frequent in high-risk patients (40.4% vs. 17.4%, *p* = 0.004). In contrast, incomplete/complete RBBB (5.0% vs. 2.2%, *p* = 0.687), RSR’ pattern (13.0% vs. 10.9%, *p* = 0.695), and T-wave abnormalities (1.2% vs. 0%, *p* = 1.000) were not different between groups. Laboratory results are presented in Table 3. White blood cell count (7.8 [6.6–9.5] vs. 8.0 [6.2–10.0] × 10^3^/µL, *p* = 0.742), hemoglobin (13.0 [12.0–14.2] vs. 13.0 [12.0–15.0] g/dL, *p* = 0.960), platelets (272 [242–308] vs. 256 [225–305] × 10^3^/µL, *p* = 0.073), and creatinine (0.80 [0.70–0.96] vs. 0.78 [0.67–0.90] mg/dL, *p* = 0.132) were similar. Lipid parameters, including triglycerides (119 [86–168] vs. 108 [80–159] mg/dL, *p* = 0.437), total cholesterol (167 [138.5–200] vs. 166 [140–194] mg/dL, *p* = 0.835), HDL cholesterol (44 [38–50] vs. 40 [35–46] mg/dL, *p* = 0.088), and LDL cholesterol (98 [74–123] vs. 104 [70–130] mg/dL, *p* = 0.872), showed no differences. Notably, HbA1c was higher in low-risk patients (5.8 [5.4–6.5]% vs. 5.5 [5.2–5.8]%, *p* = 0.475). On univariate logistic regression (Table 4), smoking (OR 2.60, 95% CI 0.87–7.80, *p* = 0.088), hyperlipidemia (OR 2.11, 95% CI 0.83–5.37, *p* = 0.116), fasting glucose (OR 1.003, 95% CI 0.992–1.013, *p* = 0.088), and SPAP (OR 0.81, 95% CI 0.62–1.07, *p* = 0.089) showed borderline associations. Significant predictors included spontaneous color Doppler shunt (OR 2.55, 95% CI 1.23–5.28, *p* = 0.018), bubble shunt at rest (OR 1.16, 95% CI 0.59–2.29, *p* = 0.012), lipomatous hypertrophy (OR 0.24, 95% CI 0.07–0.79, *p* = 0.718), and crochetage R wave (OR 3.22, 95% CI 1.41–7.34, *p* < 0.001). In the multivariate model (Table 5), independent predictors of high-risk PFO anatomy were crochetage R wave (adjusted OR 32.39, 95% CI 2.64–397.66, *p* = 0.007), lipomatous hypertrophy (adjusted OR 0.095, 95% CI 0.013–0.714, *p* = 0.022), spontaneous color Doppler shunt (adjusted OR 5.37, 95% CI 1.09–26.42, *p* = 0.039), and age (adjusted OR 0.899, 95% CI 0.821–0.983, *p* = 0.020). To address class imbalance, we performed a sensitivity analysis using repeated 1:1 down-sampling (500 iterations), which yielded a consistent direction of effect for crochetage R wave (median OR ≈ 30.2, 95% percentile interval: 2.3–very high). Although convergence issues led to wide upper bounds and variability in *p*-values, the robustness of crochetage R wave as a marker of high-risk PFO anatomy was preserved.

Furthermore, exploration of individual PFO risk score components demonstrated that patients with a crochetage R wave were more likely to have a large shunt (≥20 bubbles: 97.2% vs. 82.0%, *p* = 0.002), whereas tunnel length, tunnel angle, and atrial septal aneurysm were not significantly different between those with and without crochetage R wave (all *p* > 0.05, Supplemental Table S1).

## 4. Discussion

The primary conclusion drawn from this study is that ECG manifestations, particularly the crochetage R wave, are closely associated with the anatomical severity of PFO as defined by TEE. This is the first study to directly compare high-risk and low-risk PFO groups, demonstrating that surface ECG findings vary significantly according to anatomical risk. Moreover, the presence of a crochetage R wave was strongly related to the extent of right-to-left shunting, as reflected by bubble count, further supporting its pathophysiological relevance. In our cohort, crochetage R wave independently predicted high-risk PFO anatomy, underscoring the potential of ECG as a simple, noninvasive adjunct to imaging for refining risk stratification in cryptogenic stroke patients considered for closure.

PFO represents an embryologic remnant resulting from incomplete postnatal fusion of the septum primum and septum secundum. While physiologically important in fetal circulation, it may persist into adulthood as a potential pathway for right-to-left shunting. Autopsy and echocardiographic studies indicate that PFO is common, with an estimated prevalence of approximately one quarter of the general population, gradually declining from nearly 35% in younger individuals to about 20% in the elderly. Although often clinically silent, PFO has been linked to several conditions including cryptogenic stroke [10], decompression illness [11], migraine [12], and high-altitude pulmonary edema [13], largely through its role as a conduit for paradoxical embolism [14]. Percutaneous closure of PFO provides an effective and safe strategy to prevent recurrent stroke in appropriately selected patients [15,16]. In our study, all of patients underwent PFO closure following an ischemic cerebrovascular event, with 198 individuals (95.7%) presenting with stroke and 9 (4.3%) with TIA.

The relationship between PFO anatomy and its clinical consequences has been increasingly emphasized in the recent literature. Several studies have highlighted that anatomical characteristics of the PFO—such as canal width, length, height, and the presence of septal aneurysm—play a pivotal role in determining both shunt size and the risk of ischemic neurological events. Similarly, Węglarz and colleagues reported that wider PFO canals and larger shunt grades independently predicted stroke or TIA, underscoring the prognostic relevance of atrial septum anatomy in cryptogenic events [17]. Beyond anatomical correlates of shunting, prospective randomized data reinforce the importance of risk stratification based on PFO anatomy. The DEFENSE-PFO trial showed that patients with high-risk anatomical features—defined as large PFO size, atrial septal aneurysm, or marked septal hypermobility—derived significant benefit from closure compared to medical therapy alone, with markedly lower recurrence of stroke [18]. Taken together, these findings emphasize that not all PFOs carry equal clinical weight; rather, anatomical severity is a critical determinant of both pathophysiological mechanisms and therapeutic benefit. In line with this concept, Nakayama et al. proposed a dedicated scoring system incorporating long-tunnel PFO, hypermobile septum, Eustachian valve/Chiari’s network, large Valsalva-induced shunt, and low-angle PFO, showing that the presence of two or more features strongly predicted cryptogenic stroke [2]. We adopted these risk criteria in our study to classify patients into high-risk (score ≥ 2) and low-risk (score 0–1) groups, thereby enabling a more refined comparison of ECG correlates of PFO severity.

Recent studies have explored the potential role of ECG in identifying patients with PFO, particularly those at increased risk for ischemic stroke. Among the reported abnormalities, the crochetage R wave and RBBB have been frequently described, with some studies suggesting significant associations with the presence of PFO [5,7]. However, other investigations have failed to demonstrate consistent or statistically significant differences, highlighting the ongoing uncertainty [8]. Overall, while ECG findings may provide useful clues, there remains no consensus regarding their diagnostic reliability or predictive value for PFO. Taken together, the literature suggests that surface ECG, despite its limited sensitivity, may offer diagnostic insights when interpreted in the appropriate clinical context. The crochetage R wave, in particular, appears to be the most reproducible marker, with relatively high specificity for PFO. Nonetheless, prior work has largely been restricted to reporting prevalence, without systematically addressing the interplay between anatomical severity, shunt burden, and ECG changes. Our study builds upon this evidence by directly addressing this gap.

Our findings demonstrate that ECG changes, particularly the crochetage R wave, are not only more common in patients with PFO but also strongly related to bubble shunt count. This association reflects the underlying anatomical severity of PFO, as more complex anatomies tend to generate larger right-to-left shunts, which in turn manifest as measurable ECG alterations. In line with this, Kalesi et al. demonstrated that PFO height was significantly associated with the degree of right-to-left shunting, suggesting that specific structural dimensions can directly influence embolic potential [19]. The pathophysiological mechanism underlying the crochetage R wave may involve both chronic right atrial volume/pressure overload and localized septal conduction disturbances. Large and persistent right-to-left shunting can impose subtle remodeling of the atrial conduction system, leading to delayed or fragmented depolarization and producing the characteristic notching of the R wave in inferior leads [20]. Alternatively, excessive septal mobility or aneurysmal deformation may disrupt conduction pathways across the interatrial septum. Both mechanisms likely coexist, explaining why crochetage correlates so strongly with high-risk anatomical features and shunt burden. In this context, the crochetage R wave can be viewed as a simple but mechanistically meaningful ECG surrogate of complex anatomical and hemodynamic alterations in patients with PFO. Similarly, recent cohort data demonstrated that patients with large right-to-left shunts have a significantly higher risk of recurrent ischemic stroke compared with those with small shunts [21]. Thus, our results suggest a cascade: anatomical complexity → increased shunting → ECG manifestation. This novel observation positions ECG as an indirect marker of anatomical risk, providing a simple, noninvasive means to complement imaging in stratifying cryptogenic stroke patients considered for PFO closure. Notably, spontaneous Doppler shunting was more prevalent in the high-risk group and remained significant in both univariate and multivariate regression analyses, further underscoring the pivotal contribution of shunt burden to these pathophysiological links.

Lipomatous hypertrophy was observed more frequently in the low-risk PFO group. This phenomenon likely reflects its impact on atrial septal anatomy. By accumulating fatty tissue within the interatrial septum [22] lipomatous hypertrophy can reduce both the effective tunnel width and its longitudinal extension. Such structural alterations may lead to a more compact and less mobile septal configuration, thereby limiting the degree of right-to-left shunting. In this context, the presence of lipomatous hypertrophy may act as a protective anatomical feature, mitigating embolic potential and contributing to the relatively benign profile of PFOs categorized as low risk. Importantly, this association was confirmed in both univariate and multivariate regression analyses in our study, supporting its independent role in PFO risk stratification. Nevertheless, the precise mechanistic pathways underlying this relationship remain speculative, and further dedicated studies are warranted to clarify the role of lipomatous hypertrophy in modulating PFO anatomy and clinical risk.

## 5. Limitations

Several limitations of this study should be acknowledged. First, its retrospective, single-center design may limit the generalizability of our findings, as patient selection and management practices could differ across institutions. Second, we only assessed baseline ECG, without longitudinal follow-up to determine whether dynamic changes in ECG patterns occur after closure or during long-term monitoring. Third, the study population consisted exclusively of patients undergoing PFO closure after cerebrovascular events, which may introduce selection bias and restrict extrapolation to asymptomatic individuals or those managed conservatively. Also, there was a substantial class imbalance between the high-risk (*n* = 161) and low-risk (*n* = 46) groups, which may potentially destabilize multivariable modeling and inflate effect estimates. To address this concern, we performed sensitivity analyses using downsampling and bootstrap resampling, both of which confirmed the robustness of the association between crochetage R wave and high-risk PFO anatomy. Nevertheless, external validation in larger and more balanced cohorts remains necessary. Additionally, although multivariate modeling was performed, residual confounding by unmeasured clinical or anatomical factors cannot be fully excluded.

## 6. Conclusions

In this retrospective study of patients with cryptogenic stroke undergoing PFO closure, we found that ECG findings, particularly the crochetage R wave, were strongly associated with high-risk anatomical features as defined by TEE. Crochetage R wave emerged as an independent predictor of high-risk PFO anatomy and was also significantly correlated with the presence of a large right-to-left shunt (≥20 bubbles), underscoring its pathophysiological relevance. These findings suggest that the underlying mechanism of these ECG changes is driven by the hemodynamic and electrical consequences of larger shunt size, which imposes greater right atrial and ventricular load and alters atrial conduction. Furthermore, spontaneous Doppler shunting correlated with high-risk anatomy, reinforcing the central role of shunt burden in the pathophysiological cascade. Interestingly, lipomatous hypertrophy of the interatrial septum was more frequent in low-risk cases and may represent a protective structural factor. Taken together, these findings suggest that surface ECG may provide valuable insights into the anatomical and functional severity of PFO. Prospective, multicenter studies with larger cohorts and long-term follow-up are warranted to validate these results and to further clarify the role of ECG in guiding PFO risk assessment and therapeutic decision-making.

## Figures and Tables

**Table 1:** Baseline demographic, and clinical findings per study group.

Variable	Total (*n* = 207)	Score 0–1 (+) (*n* = 46)	Score 2–5 (*n* = 161)	*p* Value
Male Sex (*n*, %)	97 (46.9)	17 (37.0)	80 (49.7)	0.127
Age (years)	45.0 (37.0–52.0)	46.5 (41.8–53.0)	44.0 (35.5–50.09)	**0.042**
Medication (*n*, %)				
ACEi/ARB	59 (28.5)	16 (34.7)	38 (26.7)	0.183
Beta-blocker	80 (38.6)	15 (32.6)	66 (40.9)	0.392
Aspirin	42 (20.2)	13 (28.2)	29 (18)	0.188
Statin	29 (14)	10 (21.7)	19 (11.8)	0.141
Closure Indication (*n*, %)				
Stroke	198 (95.7)	43 (93.5)	155 (96.3)	0.419
TIA	9 (4.3)	3 (6.5)	6 (3.7)	0.317
Known Comorbidities (*n*, %)				
Diabetes Mellitus	34 (16.5)	9 (20.0)	25 (15.5)	0.475
Hypertension	66 (32.0)	18 (40.0)	48 (29.8)	0.195
Coronary Artery Disease	20 (9.7)	4 (8.9)	16 (9.9)	1.000
Prior Stroke	13 (6.3)	4 (8.7)	9 (5.6)	0.491
Smoking	36 (17.7)	4 (8.9)	32 (20.3)	0.078
Hyperlipidemia	45 (22.1)	6 (13.3)	39 (24.5)	0.110
Migraine	8 (3.9)	0 (0)	8 (5.0)	0.204
Deep Vein Thrombosis	6 (2.9)	0 (0)	6 (3.7)	0.342
Atrial Fibrillation	4 (1.9)	0 (0)	4 (2.5)	0.577

Abbreviations: see Table 1, TIA: Transient Ischemic Attack, ARB: Angiotensin receptor blockers, ACEi: Angiotensin Converting Enzyme-Inhibitors. Bold font indicates variables that reached statistical significance.

**Table 2:** This table presents the functional echocardiographic parameters of both ventricles together with the specific electrocardiographic findings of the patients.

Variable (*n*, (%) for the First 12 Variable, Median [IQR] for the Rest)	Total (*n* = 207)	Risk Score 0–1 (*n* = 46)	Risk Score 2–5 (*n* = 161)	*p* Value
ASA/Hypermobile septum	81 (39.5)	2 (4.4)	79 (49.4)	**<0.001**
Chiari/Eustachian Valve	41 (20.0)	1 (2.2)	40 (25.0)	**<0.001**
Bubble Shunt < 20	27 (13.5)	19 (41.3)	8 (5)	**<0.001**
Bubble Shunt ≥ 20	180 (86.4)	27 (58.7)	153 (95)	**<0.001**
Lipomatous Hypertrophy	17 (8.2)	6 (13.04)	11 (6.8)	**0.023**
Spontaneous Color Doppler Shunt	93 (48.4)	13 (31.0)	80 (53.3)	**0.010**
Bubble Shunt at Rest	112 (57.4)	24 (54.5)	88 (58.3)	0.659
Bubble Shunt Valsalva	194 (97.5)	45 (100)	149 (96.8)	0.590
Defective T Wave	2 (1.0)	0 (0)	2 (1.2)	1.000
Crochetage R Wave	73 (35.3)	8 (17.4)	65 (40.4)	**0.004**
Incomplete/complete RBBB	9 (4.3)	1 (2.2)	8 (5.0)	0.687
RSR Pattern	26 (12.6)	5 (10.9)	21 (13.0)	0.695
Tunnel Length (mm)	10.0 [9.0–14.0]	8.1 [7.0–9.1]	11.0 [9.8–14.0]	**<0.001**
IVC-PFO Tunnel Angle (°)	24.0 [15.4–36.0]	27.5 [18.8–37.0]	22.0 [13.4–35.0]	0.059
Tunnel Width (mm)	4.0 [3.0–5.0]	3.2 [3.0–4.9]	4.0 [3.2–5.0]	**0.030**
Septum Primum (mm)	31.0 [28.0–35.0]	35.5 [27.5–37.8]	30.0 [28.0–35.0]	0.163
Septum Secundum (mm)	20.5 [17.0–24.0]	22.5 [18.3–25.8]	20.0 [17.0–24.0]	0.190
Aortic Rim (mm)	6.0 [4.3–7.2]	6.0 [4.5–7.4]	5.9 [4.2–7.1]	0.321
Sinus Valsalva Diameter (mm)	32.0 [29.0–34.5]	31.0 [28.0–35.0]	32.0 [29.0–34.0]	0.710
Ascending Aorta Diameter (mm)	30.0 [27.0–33.0]	28.0 [25.5–34.5]	30.0 [28.0–33.0]	0.369
LA Diameter (mm)	34.0 [31.9–38.3]	33.0 [31.5–38.5]	34.0 [31.9–38.5]	0.585
RA Diameter (mm)	33.0 [29.8–37.0]	33.0 [32.0–35.5]	33.0 [29.0–37.0]	0.345
LA/RA Ratio	1.05 [0.97–1.13]	1.0 [0.94–1.08]	1.06 [1.00–1.14]	0.112
SPAP (mmHg)	25.0 [22.0–28.0]	28.0 [26.0–53.0]	25.0 [21.5–28.0]	0.105

Abbreviations: see Table 2, ASA: Atrial Septal Aneurysm, RBBB: Right Bundle Branch Block, IVC: Vena Cava Inferior, PFO: Patent Foramen Ovale, LA: Left Atrium, RA: Right Atrium, SPAP: Systolic Pulmonary Arterial Pressure. Bold font indicates variables that reached statistical significance.

**Table 3:** The laboratory findings of the patients are shown in the table.

Variable	Total (*n* = 207) (Median [IQR])	Risk Score 0–1 (*n* = 46) Median [IQR]	Risk Score 2–5 (*n* = 161) Median [IQR]	*p* Value
White Blood Cell (×10^3^/µL)	7.8 [6.6–9.5]	8.0 [6.2–10.0]	7.8 [6.6–9.1]	0.742
Hemoglobin (g/dL)	13.0 [12.0–14.2]	13.0 [12.0–15.0]	13.3 [12.0–14.2]	0.960
Platelet (×10^3^/µL)	261.0 [229.0–305.0]	272 [242–308]	256 [225–305]	0.073
Fasting glucose (mg/dL)	96.0 [86.0–114.0]	97.5 [85–117.5]	96.0 [86–114]	0.836
BUN (mg/dL)	26.0 [21.0–32.0]	28.0 [21–37]	26.0 [21–32]	0.305
Creatinine (mg/dL)	0.80 [0.69–0.90]	0.80 [0.70–0.96]	0.78 [0.67–0.90]	0.132
GFR (mL/dk/1.73 m^2^)	101.0 [88.0–112.0]	97.0 [83–110]	102 [90–112]	0.224
Total protein (g/dL)	69.0 [66.0–74.0]	7.0 [6.7–7.6]	7.0 [6.5–7.3]	0.663
Albumin (g/dL)	4.3 [4.0–4.5]	4.2 [4.1–4.5]	4.3 [4.0–4.6]	0.463
Triglyceride (mg/dL)	117.5 [85.0–167.0]	108 [80–159]	119 [86–168]	0.437
Total Cholesterol (mg/dL)	166.0 [140.0–196.0]	167 [139–201]	166 [140–194]	0.835
HDL (mg/dL)	43.0 [36.0–50.0]	40 [35–46]	44 [38–50]	0.088
LDL (mg/dL)	99.0 [74.0–124.8]	104 [70–130]	98 [74–123]	0.872
HbA1c (%)	5.5 [5.2–5.9]	5.8 [5.4–6.5]	5.5 [5.2–5.8]	0.475
Homosistein (µmol/L)	12.5 [9.8–14.9]	13.1 [10–17]	12.3 [9.6–14.6]	0.443

Abbreviations: see Table 4, BUN: Blood Urea Nitrogen, GFR: Glomerular Filtration Rate, HDL: High-Density Lipoprotein, LDL: Low-Density Lipoprotein.

**Table 4:** The table shows the univariate regression analyses.

Variable	*p* Value	Odds Ratio	Min–Max (95% CI)
Male Sex	0.129	0.594	0.303–1.164
Age	0.1	0.974	0.944–1.005
Diabetes Mellitus	0.476	0.735	0.316–1.713
Hypertension	0.197	0.637	0.321–1.264
Coronary Artery Disease	0.834	1.131	0.358–3.569
Smoking	0.088	2.603	0.869–7.801
Hyperlipidemia	0.116	2.112	0.832–5.367
Stroke history	0.418	1.802	0.433–7.505
Migraine	0.999		
Deep Vein Thrombosis	0.999		
Atrial Fibrillation	0.999		
White Blood Cell (×10^3^/µL)	0.509	0.954	0.831–1.096
Hemoglobin (g/dL)	0.680	1.015	0.945–1.090
Platelet (×10^3^/µL)	0.088	0.996	0.991–1.001
Fasting glucose (mg/dL)	0.622	1.003	0.992–1.013
BUN (mg/dL)	0.049	0.973	0.948–1.000
Creatinine (mg/dL)	0.180	0.484	0.167–1.399
GFR (mL/dk/1.73 m^2^)	0.161	1.012	0.995–1.029
Total protein (g/dL)	0.992	1.0	0.933–1.071
Albumin (g/dL)	0.677	0.981	0.894–1.075
Triglyceride (mg/dL)	0.589	1.001	0.996–1.006
Total Cholesterol (mg/dL)	0.438	1.003	0.995–1.012
HDL (mg/dL)	0.128	1.026	0.993–1.060
LDL (mg/dL)	0.966	1.0	0.990–1.010
HbA1c (%)	0.171	0.838	0.651–1.079
Homosistein (µmol/L)	0.146	0.961	0.911–1.014
Septum primum (mm)	0.302	0.956	0.877–1.041
Septum secundum (mm)	0.259	0.93	0.819–1.055
Aortic rim (mm)	0.122	0.895	0.777–1.030
Sinus valsalva diameter (mm)	0.747	1.022	0.894–1.170
Ascending aorta diameter (mm)	0.718	1.026	0.892–1.180
Lipomatous hypertrophy	**0.018**	0.238	0.072–0.785
Spontaneous Color Doppler shunt	**0.012**	2.549	1.230–5.283
Bubble Shunt at Rest	0.660	1.164	0.592–2.288
Bubble Shunt Valsalva	0.999		
Transcranial Doppler (Ref:No)	0.208		
10 HITS	0.076	8.143	0.802–82.678
>10 HITS	0.998		
LA Diameter (apicobasal)	0.391	1.050	0.939–1.175
RA Diameter (apicobasal)	0.526	0.969	0.878–1.069
LA/RA ratio	0.089	54.115	0.548–5345.092
SPAP (mm-Hg)	0.136	0.811	0.615–1.069
Defective T Wave	0.999		
Crochetage R Wave	**0.006**	3.216	1.410–7.338
Incomplete/complete RBBB	0.426	2.353	0.287–19.316
RSR Pattern	0.695	4.492	0.437–3.464

Bold font indicates variables that reached statistical significance.

**Table 5:** Multivariate logistic regression analysis identifying independent predictors distinguishing high-risk from low-risk PFO.

Variable	*p* Value	Odds Ratio	Min (95% CI)	Max (95% CI)
Crochetage Rw	**0.007**	32.390	2.638	397.663
Lipomatous hypertrophy	**0.022**	0.095	0.013	0.714
Spontaneous color Doppler shunt	**0.039**	5.373	1.093	26.419
Homocysteine	0.187	0.948	0.876	1.026
Age	**0.020**	0.899	0.821	0.983

Bold font indicates variables that reached statistical significance.

## Data Availability

The original contributions presented in this study are included in the article/Supplementary Materials. Further inquiries can be directed to the corresponding authors.

## Supplementary Materials

The following supporting information can be downloaded at: https://www.mdpi.com/article/10.3390/jcm14207138/s1, Table S1: Association between individual components of the PFO risk score and the presence of a crochetage R wave. Figure S1: Flow diagram of patient selection, exclusions, and risk classification in the PFO cohort.
