# Supplementary material for: Electrocardiographic Predictors of High-Risk Patent Foramen Ovale Anatomy Defined by Transesophageal Echocardiography
Source: J Clin Med. 2025 Oct 10;14(20):7138. doi: 10.3390/jcm14207138 (PMC12563818; doi:10.3390/jcm14207138)

Supplemental Table S1: Association between individual components of the PFO risk score and the presence of a crochetage R wave.

Variable	Crochetage R (-)	Crochetage R (+)	P Value
ASA / Hypermobile Septum			
Yes	81 (61.4%)	43 (58.9%)	
No	51 (38.6%)	30 (41.1%)	0.730
Chiari / Eustachian Valve			
Yes	106 (80.3%)	58 (79.5%)	0.884
No	26 (19.7%)	15 (20.5%)	
Bubble Shunt			
<20	24 (18.0%)	2 (2.8%)	
≥20	109 (82.0%)	70 (97.2%)	0.002
Tunnel Length (>10mm)	11.1±3.9	11.7±3.8	0.177
IVC-PFO Tunnel Angle (°)	26.0±15.3	27.1±15.8	0.793

Supplemental Figure S1: Flow diagram of patient selection, exclusions, and risk classification in the PFO cohort.

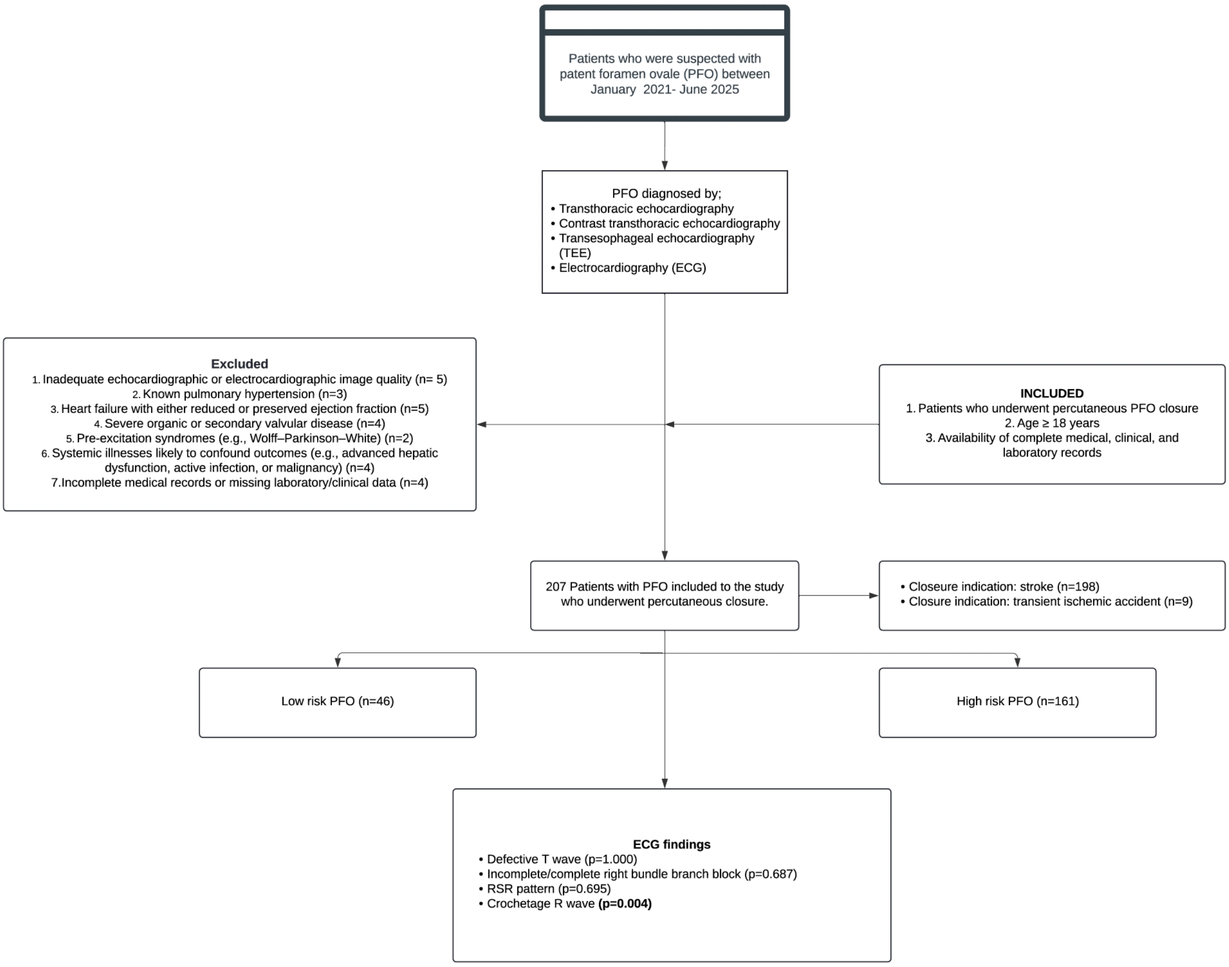

Supplement: Supplementary file 1 [file jcm-14-07138-s001.zip › jcm-3872069-supplementary.pdf]
